# Task-dependent functional connectivity changes in response to varying levels of social support

**DOI:** 10.1192/bjo.2024.742

**Published:** 2024-10-03

**Authors:** Birce Begum Burhanoglu, Ozgul Uslu, Burcu Ozkul, Kaya Oguz, Seda Eroglu-Koc, Gozde Kizilates-Evin, Cemre Candemir, Yigit Erdogan, Defne Dakota Mull, Omer Kitis, Ali Saffet Gonul

**Affiliations:** SoCAT Lab, Department of Psychiatry, School of Medicine, Ege University, Turkey; and Department of Neuroscience, Health Sciences Institute, Ege University, Turkey; School of Nursing and Midwifery, La Trobe University, Australia; SoCAT Lab, Department of Psychiatry, School of Medicine, Ege University, Turkey; and Department of Computer Engineering, Izmir University of Economics, Turkey; SoCAT Lab, Department of Psychiatry, School of Medicine, Ege University, Turkey; and Department of Psychology, Faculty of Letters, Dokuz Eylul University, Turkey; SoCAT Lab, Department of Psychiatry, School of Medicine, Ege University, Turkey; Neuroimaging Unit, Hulusi Behcet Life Sciences Research Laboratory, Istanbul University, Turkey; and Department of Neuroscience, Aziz Sancar Institute of Experimental Medicine, Istanbul University, Turkey; SoCAT Lab, Department of Psychiatry, School of Medicine, Ege University, Turkey; and International Computer Institute, Ege University, Turkey; SoCAT Lab, Department of Psychiatry, School of Medicine, Ege University, Turkey; and Department of Radiology, School of Medicine, Ege University, Turkey; SoCAT Lab, Department of Psychiatry, School of Medicine, Ege University, Turkey

**Keywords:** functional magnetic resonance imaging, generalised psychophysiological interaction analysis, social exclusion, social support, network connectivity

## Abstract

**Background:**

Having social support improves one's health outcomes and self-esteem, and buffers the negative impact of stressors. Previous studies have explored the association between social support and brain activity, but evidence from task-dependent functional connectivity is still limited.

**Aims:**

We aimed to explore how gradually decreasing levels of social support influence task-dependent functional connectivity across several major neural networks.

**Method:**

We designed a social support task and recruited 72 young adults from real-life social groups. Of the four members in each group, one healthy participant (18 participants in total) completed the functional magnetic resonance imaging (fMRI) scan. The fMRI task included three phases with varying levels of social support: high-support phase, fair phase and low-support phase. Functional connectivity changes according to three phases were examined by generalised psychophysiological interaction analysis.

**Results:**

The results of the analysis demonstrated that participants losing expected support showed increased connectivity among salience network, default mood network and frontoparietal network nodes during the fair phase compared with the high-support phase. During the low-support phase, participants showed increased connectivity among only salience network nodes compared with the high-support phase.

**Conclusions:**

The results indicate that the loss of support was perceived as a threat signal and induced widespread increased functional connectivity within brain networks. The observation of significant functional connectivity changes between fair and high-support phases suggests that even a small loss of social support from close ones leads to major changes in brain function.

Maintaining relationships with others is essential for humans because, as social beings, we have a fundamental need to interact with each other. These social interactions with others play a crucial role in the general well-being of individuals. Studies have shown that social support – ‘having or perceiving to have close ones who can provide help or care, particularly during times of stress’^1^ – improves health outcomes^2^ and self-esteem,^3^ and buffers the negative impact of stressors.^4^ Moreover, social support might be contributing to resilience to depression through stress reduction^4^ and improved self-esteem.^3^ On the other hand, lack of social interactions and social exclusion from one's group or ostracism negatively affects mental well-being, causes stress and increases vulnerability to depression.^1^ Ostracism is experienced as social pain, defined as ‘the painful feelings following social rejection or social loss’.^5^ A recent study found that even a minimal amount of support after being rejected can help to reduce or eliminate the negative emotional effects of social rejection.^6^ Social group support may become more important in competitive situations because individuals expect biased support from fellow group members, which helps them gain an advantage. In the case of not obtaining the expected support, members may even interpret their group's fair behaviours to out-groups as support loss or ostracism.^7^ Although research has explored the relationship between social support and well-being, this relationship's impact on brain network activity remains underexplored.

## Neuroimaging in social support

Neuroimaging studies have been conducted to enhance our knowledge about how the brain functions in the presence or absence of social support. Higher levels of perceived social support were associated with increased resting-state functional connectivity among default mode network (DMN) regions, such as the posterior cingulate cortex (PCC), medial prefrontal cortex (mPFC), lateral parietal cortex and inferior parietal cortex.^8^ To examine the neural responses underlying the effect of social exclusion, neuroimaging studies have been conducted with the Cyberball game.^9^ An early seminal study showed that the dorsal anterior cingulate cortex is active during the social exclusion phase of the game,^10^ and it was the region of interest for many following studies. However, a meta-analysis of Cyberball studies has failed to show consistent recruitment of the dorsal anterior cingulate cortex, but has shown increased activity in regions such as the ventral anterior cingulate cortex and PCC, inferior and superior frontal gyri, and posterior insula during social exclusion.^11^ In a recent study, when participants were subject to ostracism, they showed hyperactivation of the dorsomedial and lateral prefrontal cortex, thalamus and putamen, and increased task-based functional connectivity between the medial-frontal–striatal–thalamic regions with the ventrolateral prefrontal cortex, insula, dorsal cingulate cortex and postcentral gyri.^12^ Although studies have explored the association between social support and brain activity or connectivity, evidence on task-dependent brain connectivity is still limited. Task-dependent connectivity research can identify the coactivations of regions responding to social support and exclusion, allowing us to understand the neural mechanisms behind a wide range of cognitive functions such as the emotional processing of our own and others’ mental states, predicting their actions and acting accordingly. These abilities are essential for healthy interpersonal relationships, which have a positive effect on an individual's well-being and support handling stressful situations, which in turn, increases resilience.^4^ Since it has been shown that compared with healthy people, the brains of people with depression and bipolar disorder react differently to social exclusion,^13,14^ investigating how brain regions coactivate during social support in healthy people might help us to understand the neurophysiology behind resilience.

Overall, when previous research findings are considered in terms of brain networks, the regions associated with social support or exclusion are mainly the nodes of the DMN. However, the salience network, which comprises regions such as the anterior cingulate cortex, anterior insular (aINS) and lateral parietal cortex,^15^ and frontoparietal network (FPN) regions such as the prefrontal cortex and posterior parietal cortex (PPC), also seem to be involved.^16^ The interplay between the DMN and the salience network has already been shown in relation to social cognition. Specifically, the DMN is associated with understanding others’ mental states, whereas the salience network is associated with understanding others’ emotional states.^15^ The salience network is also known as the neural alarm system, responsible for detecting salient changes within social contexts and affective processing.^9^ The FPN includes regions responsible for cognitive and emotional control, and is critical for actively processing information and coordinating behaviour in an instant goal-driven manner, using input from other brain networks like the salience network.^16^ The salience network functions as a dynamic switch between the DMN and FPN.^17^ Thus, it is intuitive to expect all three networks’ involvement in social support and exclusion situations in which people may try to process their and other's mental/emotional states and actions, and make fairness evaluations.

## The present study

In this study, unlike most other studies, we designed a social support task by recruiting participants along with their real-life social groups, to create a more accurate representation of real-life social situations. Our task allowed participants to experience gradually decreasing levels of social support (high-support phase (HSP), fair phase, low-support phase (LSP)) by their groups. Building on previous findings and considering the involvement of different network regions, we hypothesised that there would be increased connectivity levels between the DMN, salience network and FPN regions during the LSP compared with the other phases. We anticipated a stronger difference between the LSP and HSP, where the difference in perceived social support is greater than the difference between the fair phase and HSP. Additionally, because of its higher ecological validity, we expected a more robust neurological response than that produced by the conventional paradigms applied in prior research.

## Method

### Participants

After the Institutional Ethics Committee for Medical Studies approved the study (approval date 18 August 2014, approval number 14-7/17), we recruited participants by handing out fliers on the university campus and emailing student groups. The fliers and electronic invitations allowed only four-member groups to apply for the study. We preferred university students because of the complex nature of the functional magnetic resonance imaging (fMRI) task. More than 50 groups applied to the study; however, only 20 groups (80 university students) met the study criteria. Two groups were excluded because of compliance problems during the fMRI scan. Finally, 18 groups, including 72 participants in total, constituted the study sample. Written informed consent was obtained from all individual participants included in the study.

Each member of the groups identified themselves as close friends; they had known each other for more than a year, and they rated the perceived strength of their friendship at least seven out of ten points (self-report measure through the visual analogue scale designed by the experimenter: ‘On a scale from one to ten, how would you rate the strength of your friendship?’) during the interviews. From each group, one participant who met the inclusion and exclusion criteria was randomly chosen by the experimenter to compete in the fMRI game (total 18 participants; ten women; mean age 21.72 ± 1.60 years). The inclusion criteria were being 18–25 years old and being right-handed. The exclusion criteria were having (a) a history of present or past psychiatric illness, (b) an unstable medical disease (e.g. diabetes mellitus, hypertension, etc.), (c) any first-degree relative with bipolar or psychotic disorders, (d) a history of head trauma with loss of consciousness and (e) low self-esteem score obtained from the Rosenberg Self-Esteem Scale (<15). The experiment was described to the participants as a competition between home and rival local universities in a guessing game to study students’ visuospatial abilities. They were informed that another friend group of four people (one rival to compete with, three jury members to rate the competitors’ performance) from the rival university were also participating. Each participant was told they would be judged by the members of their own team as well as the jury members of the opponent's team. However, just before the fMRI scanning, they were told that only two of the rival's friends would rate their performance because one of their friends could not come due to a traffic jam. This allowed the participant to think that the jury consists of mostly their own friends (three of five).

### Psychometric assessment

Before inclusion in the study, we interviewed all participants for their sociodemographic background and screened them with the Turkish version of the SCL-90^18^ for all possible psychiatric symptoms. We applied the Turkish version of the Rosenberg Self-Esteem Scale^19^ to exclude participants with low self-esteem levels, to reduce the risk of rapid demoralisation in the latter part of the game when the participants are competing without support.

Participants who met the inclusion/exclusion criteria completed the Turkish version of the Multidimensional Scale of Perceived Social Support before the fMRI game session to measure the level of perceived social support. The scale comprises 12 items and measures perceived social support from three distinct sources: family, friends and significant others. Each item is scored on a scale from 1 (very strongly disagree) to 7 (very strongly agree).^20^ After the fMRI scanning and before the group members reconvened, all four members also filled the Turkish version of the Need-Threat scale based on their experiences during the fMRI task. This scale is often used in Cyberball studies, and it measures the subjective experiences of the person during the experiment and consists of subscales measuring belonging, self-esteem, meaningful existence and control levels.^21^ They were instructed to separately rate the items of the Need-Threat Scale according to their feelings during the task once for where they felt like they won more and once for where they felt like they lost more. They verbally described the initial part of the game as the winning period, and the last part of the game as the losing period.

### fMRI task stimuli and procedure

#### fMRI task stimuli

**Fig. 1:**
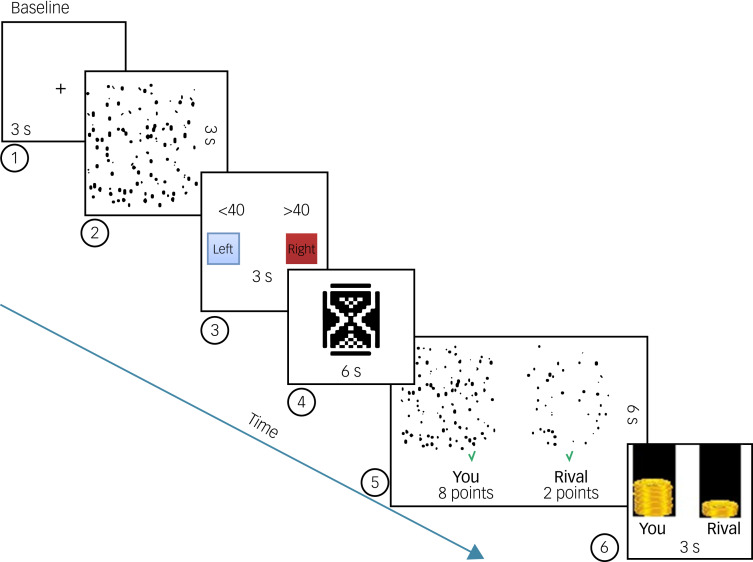
Task outline. In this example, although both competitors had given correct answers (green ticks), the participant won because of a biased point distribution by the jury. Step 1, baseline; step 2, computation; step 3, guessing; step 4, waiting (for the jury's response); step 5, feedback (assessment of their own and their competitor's performance and the jury's decision); step 6, win-loss status.

For the fMRI task, we created images including squares, hexagons, circles and stars, with different numbers and distributions (Fig. 1, second step). Each image was designed to have 30, 40 or 50 squares with varying numbers of other shapes. During the task, the participants were instructed to make a guess about the number of squares in a presented image; to guess whether there were <30/>30, <40/>40 or <50/>50. These images were also used to create 130 image pairs to be used in the feedback condition of each trial (see fMRI task procedure section). We conducted a pilot study with 30 university students to choose image pairs of different difficulty levels in terms of discriminating the number of squares. We decided to use 75 image pairs that were evaluated as high and low difficulty according to the pilot study. All stimuli were presented by a desktop computer screen projected to an MRI-compatible monitor reflecting on the mirror over the head coil in the scanner. The participants responded via a response grip placed under both of their thumbs. Software Presentation (version 19 for Windows, Neurobehavioral Systems, Inc., Berkeley, CA; http://www.nitrc.org/projects/presentation) was used to present the stimuli.

#### fMRI task procedure

The fMRI task consisting of 75 trials was completed in one single session. Each trial started with the baseline step in which the participants saw a black fixation cross on a white screen for 3 s. In the computation step, the participants viewed an image of varying numbers of geometrical shapes for 3 s. Then, in the guessing step, they were expected to guess the number of squares in the image within the following 3 s, by using the MRI-compatible response grip. The participants were warned beforehand that if they exceed the time limit to give a response, the computer will randomly choose an answer. They were also reminded that if they miss more than three consecutive responses, their team would be dismissed automatically. In the waiting step, the participant waited for 6 s for the jury's response while seeing an hourglass, and thought that all jury members, including their friends, could view and assign scores to their answer. In the feedback step, the participants observed their own and their competitors’ images and the points that the jury gave for 6 s. A green tick below the images indicated a correct guess, whereas a red cross signified a wrong guess. The jury split 10 points between their participating friend and their competitor, such as 8/2, 7/3, 6/4 or 5/5, according to the difficulty level of each image and the answers of the competitors. The jury had the freedom to give high points to a competitor for an incorrect answer to a very difficult image. This allowed the participants to assess the relative difficulty of both their own and their competitors’ images, evaluate their own performance and determine if the jury's decisions were fair or biased. The high number of geometrical shapes made the participants more dependent on their friends’ decisions for wins by reducing their confidence in their answers. Finally, in the win-loss status step for 3 s, the participants saw money bars which represented the total amount they and their competitor earned based on the judgement of their peers. Overall, each trial had the following six steps: baseline (3 s), computation (3 s), guessing (3 s), waiting (6 s), feedback (6 s), win-loss status (3 s) (Fig. 1). The total duration of the task was 30 min and 42 s.

Although the friends of the competitor assigned scores to both their friend and the competitor, predetermined scores were used during the actual experiment, to manipulate the level of social support provided to the competitor. The overall experiment was divided into three phases, with each phase consisting of 25 trials including the six aforementioned steps. During the initial 25 trials of the game (HSP), the participants obtained a higher number of points in 80% of the trials. In the subsequent 25 trials (fair phase), the participants achieved higher points in 48% of the trials. Finally, in the last 25 trials (LSP), the participants managed to get more points in only 20% of the trials. This fixed order of the phases allowed us to discover the effect of gradually decreasing levels of social support.

### fMRI image acquisition

MRI scanning was performed by a Siemens Magnetom Verio, Numaris/4, Syngo MR B17 MR scanner with a 12-channel head coil (Erlangen, Germany) in a 3 Tesla MR unit, at Ege University Hospital. First, structural images were acquired with T2-weighted axial TSE and coronal 3D-SPACE FLAIR (Dark Fluid) for any possible pathology, and T1-weighted 3D-MP-RAGE sequence (repetition time: 1900 ms, echo time: 2.5 ms, flip angle: 9°, matrix size: 256 × 256 mm, resolution: 1 × 1 × 1 mm, 176 axial slices) for co-registration with functional images. Then, fMRI data were acquired using a T2*-weighted echo-planar imaging sequence (repetition time: 3000 ms, echo time: 30 ms, flip angle: 60°, matrix size: 64 × 64 mm, resulting voxel size: 3 × 3 × 3.75 mm, 1 mm gap, 37 axial slices). A total of 614 volumes per participant per run were collected.

### Behavioural analysis

Age, gender, Rosenberg Self-Esteem Scale score and Multidimensional Scale of Perceived Social Support score variables were analysed with number, percentage, mean and s.d. (Table 1). We used a Wilcoxon test to compare the belonging, self-esteem, meaning of existence and control subscale scores of the Need-Threat Scale between the winning and the losing period (based on participants’ verbal answers). We also used a Kruskal–Wallis test to examine the main effects of task phases (HSP, fair phase and LSP) on the reaction times (average time across trials). Data analysis was performed with SPSS version 22.0 for Windows (https://www.ibm.com/products/spss-statistics).

**Table 1:** Demographic and clinical characteristics of the participants

Variable	Value			
Age, years^a^	21.72 ± 1.60			
Gender^b^	Male	Female
	8 (44.44)	10 (55.55)
MSPSS^a^	65.12 ± 14.12			
Rosenberg Self-Esteem Scale^a^	21.61 ± 3.63			
Need-Threat Scale^a^	Winning period	Losing period	*z*	*P-*value
Belonging	18.78 ± 3.70	14.56 ± 5.02	−3.08	0.002
Self-esteem	19.61 ± 3.57	13.00 ± 3.97	−3.42	0.001
Meaningful existence	23.28 ± 14.05	15.44 ± 4.23	−3.07	0.002
Control	15.22 ± 3.80	12.78 ± 3.84	−2.89	0.004

MSPSS, Multidimensional Scale of Perceived Social Support.

a. Mean ± s.d.

b. *n* (%).

### Functional connectivity analyses

#### Preprocessing

The fMRI data preprocessing was carried out with the CONN toolbox (version 21.a), running under Matlab (version R2021b for Windows, The MathWorks, Inc., Natick, Massachusetts; https://www.mathworks.com).^22^ Preprocessing steps included the standard pipeline in the following order: functional images were spatially realigned to the first volume; slice timing correction was performed for each set of functional volumes; functional and structural images were segmented to cerebrospinal fluid, grey matter and white matter, then normalised to a standard template based on Montreal Neurological Institute space and smoothed with an 8 mm full width at half-maximum Gaussian kernel.

Before the first-level estimation of functional connectivity, we estimated outlier volumes based on the motion (subject-motion mm threshold 0.9) and global signal (*z*-value threshold 5) deviations, using an ART toolbox (Artifact Detection and Repair) implemented in the CONN toolbox. This variable was used for scrubbing during the denoising step. The denoising step also includes regressing out ten principal components of the white matter and cerebrospinal fluid signal; 12 principal head motion-related artifacts, by using six head motion parameters and their first derivatives; and task-related blood-oxygen-level-dependent signals. Band-pass filtering was performed (0.008-infrasound Hz) to remove noise in the data.

#### Generalised psychophysiological interaction analysis

Functional connectivity was examined with a generalised psychophysiological interaction analysis (gPPI) analysis implemented in the CONN toolbox. The gPPI is a type of task-based functional connectivity analysis that allows us to identify task-specific changes in the relationship between a seed region and relevant brain regions as a function of task conditions. The gPPI method convolves the blood-oxygen-level-dependent signal with the canonical hemodynamic response function for each condition.^23^ In the first-level analysis, we selected regions of interest (ROIs), including the DMN's, salience network's and FPN's seeds from the CONN's built-in network-based atlas, namely the mPFC (*x* = 1, *y* = 55, *z* = −3), PCC/precuneus (*x* = 1, *y* = −61, *z* = 38) and left (*x* = −39, *y* = −77, *z* = 33) and right (*x* = 47, *y* = −67, *z* = 29) lateral parietal cortex (for the DMN); the dorsal anterior cingulate cortex (*x* = 0, *y* = 22, *z* = 35), left (*x* = −44, *y* = 13, *z* = 1) and right aINS (*x* = 47, *y* = 14, *z* = 0), left (*x* = −32, *y* = 45, *z* = 27) and right (*x* = 32, *y* = 46, *z* = 27) rostral PFC, and left (*x* = −60, *y* = −39, *z* = 31) and right (*x* = 62, *y* = −35, *z* = 32) supramarginal gyrus (SMG) (for the salience network); left (*x* = −43, *y* = 33, *z* = 28) dorsolateral PFC and right (*x* = 41, *y* = 38, *z* = 30) lateral PFC, left (*x* = −46, *y* = −58, *z* = 49) and right (*x* = 52, *y* = −52, *z* = 45) PPC (for the FPN) as source and target seeds in line with the hypotheses of the study. Following this, for each participant we generated separate gPPI models for each seed. Each model contained one regressor of the seed time-series, individual regressors for each task phase (HSP, fair phase and LSP), and regressors for the seed × phase interaction. In the second-level (group-level) analysis, ROI-to-ROI analysis was used to compare connectivity between selected ROIs for the HSP versus LSP, HSP versus fair phase and LSP versus fair phase contrasts. The results were assessed with a threshold of voxel *P* < 0.001, uncorrected, in combination with a cluster *P* < 0.05, corrected for a false discovery rate of multiple comparisons.

## Results

### Behavioural results

Demographic variables and psychometric assessment scores of the group are shown in Table 1. After the fMRI scanning, all participants described the initial part of the game as the winning period, and the last part of the game as the losing period. They also reported that their support started to decrease in the middle of the game. In line with these subjective verbal answers, the scores of all subscales in the Need-Threat Scale (belonging: *z* = −3.08, *P* = 0.002; self-esteem: *z* = −3.42, *P* = 0.001; meaningful existence: *z* = −3.07, *P* = 0.002; control: *z* = −2.89, *P* = 0.004) (Table 1) decreased in the losing period compared with the winning period, suggesting that the task had a significant effect on the perception of their self-esteem and group (social) support. The reaction times of participants decreased as the task progressed (HSP: 1395 ms; fair phase: 1220 ms; LSP: 1167 ms) (*χ*^2^ = 12.2, d.f. = 1.17, *P* = 0.003).

### Task-dependent changes in ROI-to-ROI connectivity

**Fig. 2:**
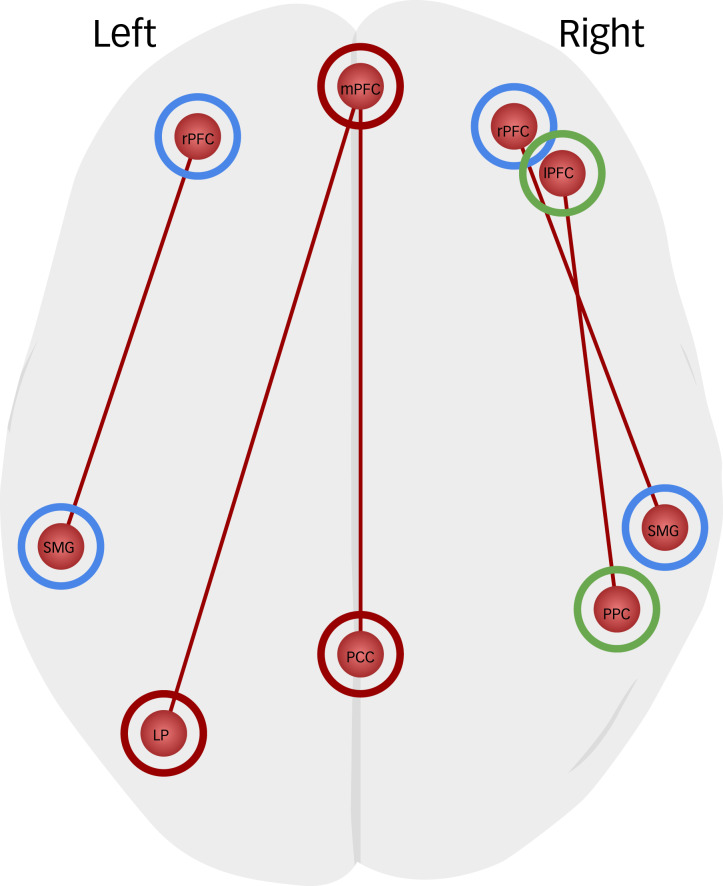
Brain regions that showed significantly increased task-dependent ROI-to-ROI connectivity during the contrast of fair phase > HSP. Blue circles indicate the salience network regions, red circles indicate the default mode network regions and green circles indicate the frontoparietal network regions. (cluster-level *P*_FDR_ < 0.05). FDR, false discovery rate; HSP, high-support phase; LP, lateral parietal cortex; lPFC; lateral prefrontal cortex; mPFC, middle prefrontal cortex; PCC, posterior cingulate cortex; PPC, posterior parietal cortex; ROI, region of interest; rPFC, rostral prefrontal cortex; SMG, supramarginal gyrus.

**Fig. 3:**
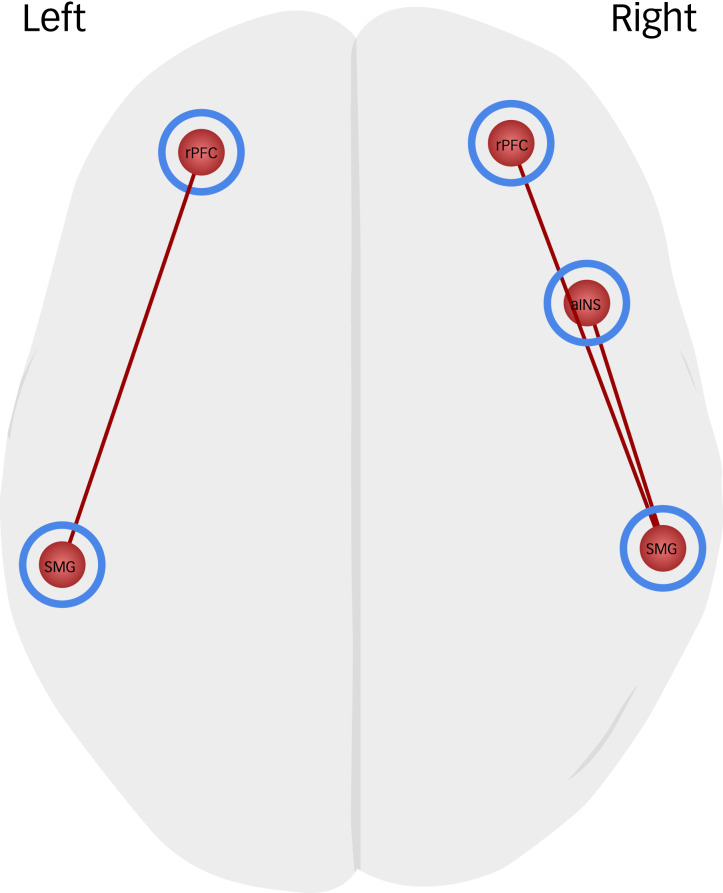
Brain regions that showed significantly increased task-dependent ROI-to-ROI connectivity during the contrast of LSP > HSP. Blue circles indicate the salience network regions, red circles indicate the default mode network regions and green circles indicate the frontoparietal network regions. (cluster-level *P*_FDR_ < 0.05). aINS, anterior insula; FDR, false discovery rate; HSP, high-support phase; LSP, low-support phase; ROI, region of interest; rPFC, rostral prefrontal cortex; SMG, supramarginal gyrus.

The results of the gPPI analysis demonstrated that participants showed increased connectivity among salience network, DMN, FPN nodes during the fair phase, in which they received a fair amount of support, compared with the HSP, in which they received a high amount of support (Table 2, Fig. 2). Based on the correlation values in each condition, the left rostral PFC had stronger connectivity with the left SMG, the right rostral PFC had more positive connectivity with the right SMG, the mPFC had more positive connectivity with the PCC and the left lateral parietal cortex, and the right lateral PFC had stronger connectivity with the right PPC. During the LSP (versus HSP), in which they received the least amount of support, participants showed increased connectivity among only salience network nodes (Table 2, Fig. 3). Based on the correlation values in each condition, the left rostral PFC had stronger connectivity with the left SMG, the right rostral PFC had more positive connectivity with the right SMG and the right SMG had more positive connectivity with the right aINS. There was no significant difference in functional connectivity between the LSP and fair phase.

**Table 2:** Regions that showed significantly increased task-dependent ROI-to-ROI connectivity between the phases

Contrast	Region 1 (MNI coordinates)	Region 2 (MNI coordinates)	*r*_HSP_	*r*_fair phase_	*r*_LSP_	*t*	*P*-value (FDR)
**Fair phase > HSP**	Left rPFC (salience network) (−32, 45, 27)	Left SMG (salience network) (−60, −39, 31)	−0.0178	0.1461	–	4.28	0.007
	Right rPFC (salience network) (32, 46, 27)	Right SMG (salience network) (62, −35, 32)	−0.1375	−0.0361	–	4.71	0.003
	mPFC (DMN) (1, 55, −3)	PCC (DMN) (1, −61, 38)	−0.0877	−0.0121	–	4.90	0.002
	mPFC (DMN) (1, 55, −3)	Left lateral parietal cortex (DMN) (−39, −77, 33)	0.0341	0.1460	–	4.51	0.002
	Right lPFC (FPN) (41, 38, 0)	Right PPC (FPN) (52, −52, 45)	−0.0528	0.0348	–	4.04	0.012
**LSP > HSP**	Left rPFC (salience network) (−32, 45, 27)	Left SMG (salience network) (−60, −39, 31)	−0.0178	–	0.1239	4.86	0.002
	Right rPFC (salience network) (32, 46, 27)	Right SMG (salience network) (62, −35, 32)	−0.1375	–	−0.0381	4.19	0.008
	Right SMG (salience network) (62, −35, 32)	Right aINS (salience network) (47, 14, 0)	0.0601	–	0.0139	4.07	0.007
**Fair phase > LSP**	–	–	–	–	–	–	–

*r* represents the correlation coefficient corresponding to each ROI-pair for each condition. ROI, region of interest; MNI, Montreal Neurological Institute; HSP, high-support phase; LSP, low-support phase; FDR, false discovery rate; rPFC, rostral prefrontal cortex; SMG, supramarginal gyrus; mPFC, middle prefrontal cortex; DMN, default mode network; PCC, posterior cingulate cortex; lPFC, lateral prefrontal cortex; PPC, posterior parietal cortex; FPN, frontoparietal network; aINS, anterior insula.

## Discussion

The current study aimed to investigate how gradual loss of social support affects brain connectivity, and provide data on how the connectivity within different brain networks changes as participants lose support from their social group. For this purpose, we designed a social support task comprising three phases of decreasing levels of social support (HSP, fair phase and LSP, respectively). We created an experimental environment where participants needed the support of real-life friend for easy wins in a social competition. We found connectivity differences between the HSP and LSP, partially supporting our hypothesis. We were expecting more regions to show increased connectivity in the LSP > HSP contrast than the fair phase > HSP contrast; however, we observed the opposite. As they first started to lose the support from their social group (fair phase compared with HSP), they showed increased connectivity within DMN, FPN and salience network nodes. Then, when they markedly lost that support (LSP compared with HSP), the connectivity differences within DMN and FPN disappeared and those within salience network connectivity came into prominence. Interestingly, we found no difference between the fair phase and LSP. The absence of connectivity differences between the fair phase and LSP, combined with the observation of significant changes between the fair phase and HSP, suggest that even a small loss of social support from close ones leads to major changes in brain function.

At the beginning of the task (HSP), participants had a predictable and secure environment because of the expected support through disproportionately positive feedback. It was an expected situation because biased behaviours to in-group members is a (social) norm in many groups.^24^ However, as the game proceeded (fair phase), the participants had unbiased feedback, which made their social environment feel unpredictable. Notably, during the fair phase compared with the HSP, increased functional connectivity between the mPFC, PCC and lateral parietal cortex (left angular gyrus) in DMN regions; lateral PFC and PPC (right angular gyrus) in FPN regions; and rostral PFC and SMG in salience network regions were recruited. Studies on social exclusion have found that it activates two neural networks: a social pain network, which is associated with distress and is characterised by increased activity in the anterior cingulate cortex and aINS; and a mentalising network, which is associated with understanding others and is characterised by increased activity in the mPFC, PCC/precuneus, and temporoparietal junction.^10,25,26^ The anterior cingulate cortex and aINS are nodes of the salience network, whereas the mPFC, PCC/precuneus and temporoparietal junction are nodes of the DMN.^17^ Our connectivity findings in similar networks indicated that receiving social support at a fair level (through unbiased feedback) might have been perceived as losing support, and might have made the participants feel distressed.

The DMN is associated with a variety of internally directed mental representations like self-reflection, autobiographical memory and future event simulation.^27^ Similar to mentalising network functions,^9^ it also has a critical role for interpersonal skills like perspective taking, empathy and discriminating one's own and the other's mind (theory of mind).^28^ Especially, mPFC and PCC activation is associated with self-referential processing, which is the cognitive process of relating information coming from the external world to the self.^29^ We observed increased functional connectivity between these two DMNs nodes during the fair phase compared with the HSP. This observation may suggest that when participants lose support of a close one, they engage in self-evaluation processes using social information related to the jury's feedback, which may be potentially related to the decreased sense of belonging, self-esteem, meaningful existence and control scores (Need-Threat Scale) that the participants in the current study reported, as they perceived a support loss. Our finding is in line with previous studies reporting that self-referential and social processes are inseparably linked,^29,30^ and suggests that self and other referential processes overlap in the mPFC and PCC.^15^ Indeed, these regions were also associated with higher levels of perceived social support.^8,31^

We also found increased connectivity during the fair phase compared with the HSP between two FPN regions: the lateral PFC and PPC (angular gyrus). The FPN is associated with specific cognitive domains like attention, and executive functions like planning, and cognitive control.^16^ Thus, during our task, when the participants lost biased feedback, FPN activity was expected because the FPN evaluates incoming unpredictable information through further higher-order processing.^32^ Our finding is in line with previous social exclusion research that showed involvement of the lateral PFC.^12,13,33^ The angular gyrus is a part of the temporoparietal junction, which plays a crucial role in executing theory-of-mind functions like the ability to be aware of the mental states of others.^34^ Moreover, when people receive information about possible reasons for exclusion and their need to understand what was happening is reduced, temporoparietal junction activity is also reduced.^35^ Accordingly, studies demonstrated the involvement of the angular gyrus in social cognition, information processing, attention and theory of mind.^36^ Although we did not explicitly measure it, it is possible that our participants made an effort to understand the intention of their fellow jury members when losing support, and make fairness evaluations. Thus, the increased connectivity between these regions might be explained by the participants’ attempt at understanding others.

In the last phase of the task, the initial safe and predictable environment changed to an unpredictable and insecure environment. It is to be expected that participants keep engaging the DMN and FPN during the LSP, when they feel the least amount of support. This is because relevant regions are working together by using external cues to give as accurate as possible explanations about others’ mental states,^15^ which leads to updated internal predictions to help generate appropriate responses.^16^ However, the results showed that participants engaged only the salience network during the LSP compared with the HSP. Based on recent imaging findings showing the involvement of salience network regions in social exclusion,^10^ we speculate that salience network connectivity might be a result of the participants’ levels of distress or frustration, starting at the fair phase and continuing through the LSP, although we did not assess participants’ emotional states. The reason why the participants did not show significant connectivity differences in DMN and FPN regions between the LSP and HSP could be because of their lack of motivation to understand their friend's mental states after being frustrated, or their preference for mentalising about positive social information and a tendency to avoid the negative aspects of their social environment following social exclusion.^25^ When excluded, participants may have prioritised dealing with their own negative emotions and the potential threat of further exclusion. This might have limited the resources available for higher-order processing in the DMN and FPN.^16^

Salience network activation is often associated with affective processing and the experience of social distress that indicates a difference between what is expected and what is obtained from close ones or a social group.^10,35^ The salience network is also known as the neural alarm system, responsible for detecting salient changes in the social context when being excluded and for emotionally processing aversive experiences.^9^ Therefore, it is not surprising to observe functional connectivity between these regions, which play a crucial role in experiencing social distress. Specifically, SMG-aINS connectivity was increased in the LSP. Activation of the aINS is associated with evaluating the emotional and motivational salience of specific stimuli, providing an interface between external information and internal motivational states.^37^ It is also a brain area involved in the processing of negative affect during social exclusion and self- and other-directed aversive experiences.^11,12,35^ Our findings for increased SMG-aINS functional connectivity during the LSP are therefore in line with other studies showing that aINS coactivation with salience network nodes is critical in detecting salient external stimuli and making emotional judgements.^38^ Contrary to other studies that have shown the involvement of the anterior cingulate cortex in social exclusion, we could not detect its functional connectivity with other network nodes. However, our findings could be considered consistent with the results of a recent meta-analysis that failed to show its consistent recruitment.^11^ The discrepancy between studies might be because of the different task designs, choices of ROIs and ecological validity levels.

Overall, these results indicate that the loss of support was processed as a threat signal and induced widespread increased functional connectivity within brain networks. Identifying differences in functional connectivity of network regions across loss of social support allows us to better understand neural mechanisms behind numerous cognitive functions such as the emotional processing of our own and others’ mental states, predicting their actions and acting accordingly. These abilities are essential for healthy interpersonal relationships, which have a positive effect on an individual's well-being and support handling stressful situations. Conversely, any dysfunction within network connectivity may contribute toward increasing vulnerability for disorders like depression, when being exposed to exclusion.^13^ For example, DMN activations are crucial for self–other referential processing during social interactions. Hyperconnectivity in the DMN might be the neural underpinning of atypical self–other evaluation processing such as negative self-representations and misinterpretation of other's intentions in depression through rumination.^39^ The presence of these dysfunctions during similar situations in healthy adults could be signs of vulnerability, whereas their absence could be signs of resilience.

In our study, the almost complete loss of support during the LSP (versus HSP) was not associated with DMN and FPN activity. Although we did not explicitly measure it, this might suggest that the brain functions in a compensatory way that could protect them from the stress of being socially excluded, i.e. they might be resilient. For instance, one study^40^ found a negative correlation between resilience scores and PCC (DMN) functional connectivity; similarly, another recent study^41^ showed a negative association between resilience scores and functional connectivity of PCC and angular gyrus (DMN) in a low-support group. So, the lack of significantly different DMN connectivity in our participants during social support loss could be considered consistent with the idea that they might be resilient, as the mentioned studies showed how decreased DMN connectivity was associated with higher resilience. Additionally, we observed decreasing reaction times from the HSP to LSP, which could be the result of a confounding practice effect. However, it may also be another indicator of resilience against the negative effects^1^ of social exclusion. Although there is no definite relationship between reaction time speed and motivation, studies have shown that in the presence of reward and performance feedback, faster reaction times were associated with increased motivation.^42,43^ Considering that one might expect decreased motivation levels when losing social support,^21^ and that our task included both reward and performance feedback, the fact that our participants’ reaction times decreased as they lost support could be interpreted as they did not lose their motivation, which might indicate resilience. It is crucial to examine the neural effect of social exclusion not only on healthy people, but also psychologically vulnerable/resilient groups. Exploring how brain function changes in these groups might help us to develop early intervention and further treatment strategies.

### Strengths

The main strength of our study is that unlike previous studies, we recruited participants along with their real-life social groups to create a realistic social support task that better reflected real-life social situations. The participants’ behavioural responses confirmed that manipulating the winning rates in different phases worked as intended. The scores on all subscales of the Need-Threat Scale, which includes belonging, self-esteem, meaningful existence and control, significantly decreased in the last part of the game compared with the initial part. Furthermore, to study social exclusion, most studies used the Cyberball paradigm, which has two phases: inclusion and exclusion. In the inclusion phase, participants are included in the game at a fair amount, acting as a neutral baseline to contrast with the exclusion phase. In addition to these phases, our task included an HSP. This design enabled us to contrast when perceived support is inflated and to compare the brain responses to three varying levels of social support. We made this comparison by focusing on the DMN, FPN and salience network, whereas most studies only focused on the salience network. This helped in identifying the specific neural correlates of social support loss and allowed us to conclude that even a small loss of social support from close ones leads to major changes in brain function. The order of the phases of our task were the same for all participants, which may have produced a confounding order effect. However, we believe that the benefit of being able to assess the gradual loss of support via this consistent order outweighs the potential confounding order effect.

### Limitations and future directions

The current study had several limitations. The most prominent limitation of this study is its small sample size; however, considering that we included participants with their real-life social groups, we argue that we obtained more robust results. Second, we excluded participants with low self-esteem levels to reduce the risk of rapid demoralisation in the latter part of the game when the participants should compete without support, and this might have limited the variation in brain responses and made it harder to generalise the results of the study to larger populations. Third, we only included university students aged 18–25 years. It is known that network connectivity during rest and social cognition tasks show age-related differences,^44^ and so this also limits the generalisability of our findings across different age groups. We also did not control for the effect of gender; however, the number of males and females in our sample were almost equal. Future research may consider the confounding effects of gender, individual differences in personality, resilience and past experiences when studying brain responses to loss of social support. Further research might also explore how network connectivity may be affected by differing levels of social support within clinical populations.

In conclusion, the current findings represent an important extension of the existing literature understanding the effect of social support on brain function. Even a small change in expected support leads to major changes in brain connectivity. Considering the effect of social interactions on mental health, more research on the interplay between perceived social support, brain connectivity and depression is needed to validate these findings, and develop further treatment and early intervention strategies.

## Data Availability

The data that support the findings of this study are available from the corresponding author, A.S.G., upon reasonable request.
